# Preservation of ovarian function during chemotherapy and radiotherapy in young women with malignancies

**Published:** 2014-06

**Authors:** Maryam Eftekhar, Soheila Pourmasumi, Mojgan Karimi-Zarchi

**Affiliations:** 1 *Recearch and Clinical Center for Infertility, Shahid Sadughi University of Medical Sciences, Yazd, Iran.*; 2 *Department of Obstetrics and Gynecology, Shahid Sadoughi Hospital, Shahid Sadoughi University of Medical Sciences, Yazd, Iran*

**Keywords:** *Malignancy*, *Fertility pres*ervation, Leukemia, Cervical cancer, Endometrial cancer, *Ovarian cancer*

## Abstract

Malignancies are not rare in girl and women during their reproductive years. Over the past three decades, the survival rate for cancer has been improving due to progress in cancer diagnosis and treatment. These patients frequently experience a variety of treatment, and disease-related side effects that diminish their quality of life during and after treatment; among these are loss of fertility and sexual dysfunction. There have been recent advances in the field of fertility preservation, which can allow many of these genital cancer survivors to have children in the future. This topic review discusses available options and specific strategies for fertility preservation in adolescent and young women with malignancies who wish to preserve their ability to become pregnant in the future.

## Introduction

Fertility preservation is an important part of improving the quality of life of cancer in girl or young women who have survived after cancer (1). It is recommended to encourage physicians to explain fertility issues in reproductive-age cancer patients prior to starting cancer treatments (2). The psychological problems of cancer treatment-related infertility in women who undergoing gynecologic malignancies should be considered. Many of these women expressed feelings of depression, grief, stress, and sexual dysfunction (3). Conservative surgery has been proposed to preserve the reproductive organs (4-7).

These methods allow some patients conceiving naturally. Although, the number of patients needed to use assisted reproductive techniques due to subfertility type of their problem. Indications for fertility-sparing surgery include an early stages well-differentiated low-grade tumor with low malignant potential (8). In this review, we present fertility-sparing surgical procedures that have been suggested for fertility preservation in malignancies.

**Leukemia**

Leukemia is a common hematologic disorder that needs chemotherapy and has a bad effect on ovarian function in girl and young adult or adolescents. In this center and in Iran we have minimal condition for freezing of ovarian tissue or oocyte preservation. As a matter of fact this condition is done as an experimental work in our center. But we had had a good work on preservation of ovarian function by gonadotropic releasing hormone before and during chemotherapy in patients with leukemia and lymphoma (1, 9). 

**Cervical cancer**

It is estimated that approximately 10-15% of cases of cervical cancer were diagnosed in reproductive age (10). The percentages of women surviving after surgical treatment of early stage cervical cancer are likely to be higher. Although, treatment of this cancer in young patients may leads to sterilization due to either radiation therapy or radical hysterectomy (11). In 1987, Dr. Daniel Dargent *et al *introduced a method of radical trachelectomy combined with pelvic lymphadenectomy for the treatment of selected cases of early stage cervical cancer. In this method, after resection of the cervix with parametrial tissues, and preserving of the uterus and most proximal portion of the cervix, patients will be able to become pregnant at future (12).

Patients selected for radical vaginal trachelectomy in most published series are FIGO stage IB2 or earlier with a tumor size of 2.5 cm or less and squamous cell or adenocarcinoma type. Overall, recurrence rate of 5% and death rate of 3% reported after vaginal trachelectomy (13-15). These results are similar to the percentage reported for radical hysterectomy for similarly sized lesions. Almost 40% of recurrences occur in the parametrium or pelvic sidewall, probably because of insufficient parametrial excision.or presence of microscopic lymph-vascular space invasion (LVSI), and in 25% of cases recurrences occur in the pelvic, paraaortic, and/or supraclavicular nodes (16). 

Lesions ≥2cm are associated with a higher risk of recurrence. The presence of LVSI also seems to be related with a higher risk of recurrence (12% vs. 2%). Adenocarcinomas are not clearly related with a higher risk of recurrence. however, Hertel *et al* showed 3 of the 4 recurrences were adenocarcinomas (17). Adenosquamous histology does not appear to increase recurrence rate (18). Neuroendocrine tumors of cervix are very aggressive, and fertility-preserving surgery should never propose to these patients (19). According to literature reviews, after vaginal trachelectomy, most of women became pregnant “naturally” without assisted reproductive help. Although, there were concern about shortened cervix related subfertility (*e.g.* insufficient mucus production) (14, 18, 20). 

Interestingly, between the women who had been struggling to get pregnant in Coven’s series, five had a history of infertility and three became pregnant spontaneously. Therefore, we should exclude patients with a “history” of infertility from this procedure. Nevertheless, in Dargent’ series, eight women did not get pregnant including five women with prior history of infertility, and three with unsuccessful IVF cycle (21). A present literature reviewing 256 pregnancies after vaginal trachelectomy indicates that 62% of pregnancies reach the third trimester, of which 65% will reached to term pregnancy. The preterm delivery reported in 28% of pregnant women. However, only about 12% will end with significant prematurity with gestational age less than 32 weeks. Eventually 40% of all pregnancies will lead to birth of healthy babies (18).

The important question is whether a total hysterectomy should be performed after the family complete. Mattevet and coibanu answered that because the risk of recurrence in long term study is very low, even negligible. In addition, hysterectomy after vaginal trachelectomy is a difficult procedure; the uterus does not need to be removed.. However, the approach should be modulated based on the histological type of initial lesion. Indeed, it is recognized that cervical adenocarcinomas sometime have a multifactorial character which could potentially predispose to isthmic recurrence in the patients (22-25).

A study by Anna Fagotti *et al* was suggested excision of cone as fertility-sparing treatment in 17 early- stage cervical cancer patients. In this study, none of the patients were received neoadjuvant chemotherapy and two patients were received three course of adjuvant chemotherapy. No recurrences were reported after a median follow-up of 16 months. Two of five patients (40%) attempting to conceive had a spontaneous pregnancy and delivery (23).

**Endometrial cancer**

Endometrial cancer usually occurs in postmenopausal women (24). The standard surgical staging for this cancer includes hysterectomy with bilateral salpingo-oophorectomy and lymph node biopsy (25). This treatment usually will not make problems, because endometrial cancer occurs most commonly with menopause. Approximately 3-5% of patients with endometrial cancer are younger than 45 years of age, and it is rare in women younger than 25 years (24). The diagnosis of endometrial carcinoma in young women is usually related with a hyperestrogenic state (26). 

Approximately 25% of cases occur in patients with polycystic ovarian syndrome (27). Most of young women with endometrial cancer have a well-differentiated adenocarcinoma that is usually estrogen receptor positive with no or minimal myometrial invasion (28). The prognosis in these patients is very good, and the cure rate is more than 95% (29).

There are many treatment protocols for the conservative management of endometrial cancer. Although, there is no general agreement about use of progestational agent and dosage, the duration of treatment, and the long term monitoring program (30). Synthetic progestins like megestrol acetate at 40-160 mg/day and medroxyprogesterone acetate at 200-600 mg/day have been most commonly used. Response rates differ from 83-94% for atypical hyperplasia and from 57-75% for endometrial adenocarcinoma. The length of therapy differ,s between 10 weeks to 12 months. Most complete regression appears in the first 3-6 months of management, and it is recommendeded that response to treatment be defined by dilatation and curettage at regular intervals, usually every 3- month after regression of tumor. Thereafter, women can try to conceive naturally or with assisted reproductive technologies (29-30). 

In a meta-analysis by Gallos *et al* assisted reproductive thechnology (ART) versus spontaneous pregnancy after medical treatment of endometrial cancer and atypical complex endometrial hyperplasia were evaluated. Out of 451 women that had fertility sparing treatment for endometrial cancer or atypical complex endometrial hyperplasia, 142 tried to conceive with the help of ART, and 56 of them achieved at least one live birth (around 39.4%). The remaining 309 women attempted to conceive spontaneously and 46 women achieved at least one live birth, with a rate of 14.9%. The differences between two groups was statistically significant (31). A limited number of studies have achieved on the other treatments besides oral progestins to manage endometrial cancer (32). 

Tamoxifen and gonadotropin releasing hormone (GnRH) agonists have moderate effects on advanced and recurrent endometrial cancer, but there are limited data supporting their use for early-stage, well differentiated cancers (33-35). The effectiveness of a P-containing intrauterine device (IUD) in management of well-differentiated early stage (IA) endometrial carcinoma is unclear. Its possibility was examined in a study by Montz *et al* (34). In conclusion, medical therapy alone for early-stage low-grade endometrial carcinoma in young patients has been reported in previous works. Nonetheless, complete guidelines for selection, treatment, and follow-up are not yet recognized.

**Epithelial ovarian cancers (EOCs)**

Although EOC is primarily disease of postmopausal women (35). Present data estimates that 3-17% of EOCs found in women aged less than 40 years and 7-8% of all malignant stage I epithelial tumors of the ovary occur in women aged less than35 years (36). The importance of age as a prognostic factor for ovarian cancer has been debated. early stage disease and lower grade tumors more likely to occur in younger women (37). Several reports have showed that, even after accounting for stage and grade, young women have a better prognosis than older women (38-39).

In a population-based study, young women had a significant higher survival rates across all stages. In another study, the 5-year survival rate for women with stage III and IV tumors aged <30 years was 56% vs. 22% for patients aged >60 years (40). The standard surgical protocol for epithelial ovarian cancer consists of hysterectomy in combination with bilateral oophorectomy. In young women, this treatment not only leads to the loss of reproductive function, but also predispose them to the long-term estrogen deficiency (36).

There are limited data about safety of fertility sparing surgery in EOC. Park *et al* in a 56- month follow up study of 62 young women that treated with fertility sparing surgery defined as the preservation of uterus and at least one ovary found that 11 patients developed recurrence and 6 patients died of disease. They showed that patients with stage >1C or grade III tumors had significantly worse survival. In the mentioned study, 9 patients tried to conceive and three of them reached term pregnancy. They concluded that fertility sparing surgery can be suggested for young patients with EOC who desired fertility preservation with stage IA-C and grade I-II (36). A number of studies have shown that patients with stage I but grade II or III have a high risk for recurrence. So, fertility sparing surgery recommended only in patients with stage IA and grade I (41).

**Borderline ovarian tumors**

Borderline ovarian tumors (BOTS) are characterized by a degree of cellular proliferation and nuclear atypia in the absence of stromal invasion and infiltrative destructive growth (42). BOTS are reported in 10-20% of ovarian epithelial tumors (43, 44). Most of BOTS are diagnosed in early stage of disease and have an excellent prognosis. Most of them occur in women of reproductive age. Therefore, BOTS affects women who desire for pregnancy (42). 

The standard protocol for treatment of BOTS contains total abdominal hysterectomy and bilateral salpingo-ophorectomy (43). Fertility sparing surgery is defined as preseservation of uterus and at least a part of one ovary. The results of limited detailed studies have shown that the recurrence after cystectomy is 12-15% and is similar to recurrence rate after salpingo-oophorectomy. Natural conception reported in 32-65% of patients with BOTS after conservative surgery. However, many of patient eventually need to ovulation induction or ART (24) .

**Germ cell ovarian carcinoma**

Ovarian germ cell tumors include 20-25% of all ovarian neoplasms. Although, in 3% of cases of these may become malignant. Malignant ovarian germ cell tumors often found in young women and children. Whiles, benign teratoma is the most common form of these neoplasms, which rarely underwent malignant degeneration. Malignant ovarian germ cell tumors include primitive or immature element. Most common malignant ovarian germ cell tumors include dysgerminomas, immature teratomas, yolk sac tumors, mixed germ cell tumors, embryonal carcinomas, choriocarcinomas and polyembryomas ovarian (44). Germ cell tumors response in a different manner to chemotherapy (45). Conservative surgery is standard treatment in young patients. In patients with dysgerminoma, biopsy of contralateral ovary is recommended, because there is 10% risk for occult disease (22). 

## Conclusion

Gynecologic malignancy can be destructive to young patients who desire future fertility. The standard treatments for many of these malignancies often lead to infertility. Some Options for these patients exist to preserve fertility. An excisional cone biopsy or radical vaginal trachelectomy in very select patients allows preservation of fertility in young women with cervical cancer. Medical hormone therapy is a choice for patients with grade 1 endometriosis adeno carcinomas of the uterus limited to the endometrium. Patients with ovarian cancers do not need hysterectomy or BSO. 

A multidisciplinary approach is essential; the care of these patients' needs to be coordinated with gynecologic, oncologists, endocrinologists, and perinatologists. Patients require recognizing the limited data in most Instances and the unknown risks that they have been proposed, as well as the extreme follow-up that is essential.
